# Factors Associated With Low Utilization of Cervical Cancer Screening Services in Gazipur, Bangladesh

**DOI:** 10.1155/ogi/4476955

**Published:** 2025-12-22

**Authors:** Sadia Fatema Kabir, Muhammad Ashik-Ur-Rahman, Abdur Rahman, Md. Afzal Hossen, Rowfun Rahman, Farhana Huq, Mohibbul Haque, Junnatul Fardous Marfi, Md Abdullah Saeed Khan, Mohammad Delwer Hossain Hawlader

**Affiliations:** ^1^ BRAC Health Programme, Bangladesh Rural Advancement Committee (BRAC), Dhaka, 1216, Bangladesh; ^2^ Medical Administration, Bangladesh Navy, Dhaka, 1213, Bangladesh; ^3^ Immunization and Vaccine Development Division, World Health Organization, Bangladesh, Dhaka, 1212, Bangladesh; ^4^ National Tuberculosis Control Program, Directorate General of Health Services, Dhaka, 1212, Bangladesh; ^5^ Department of Gynecological Oncology, Bangladesh Medical University, Dhaka, Bangladesh; ^6^ Department of Public Health and Informatics, Bangladesh Medical University, Dhaka, Bangladesh; ^7^ PricewaterhouseCoopers, Dhaka, Bangladesh; ^8^ Directorate General of Health Services, Dhaka, 1212, Bangladesh; ^9^ Department of Public Health, North South University, Dhaka, 1229, Bangladesh, northsouth.edu; ^10^ NSU Global Health Institute (NGHI), North South University, Dhaka, 1229, Bangladesh, northsouth.edu

**Keywords:** Bangladesh, barriers, cervical cancer, knowledge, screening, utilization

## Abstract

**Background:**

Despite its availability, cervical cancer screening services continue to remain underutilized in many regions. This study aimed to assess the prevalence and determinants of cervical cancer screening uptake among women in the north‐central area of Bangladesh.

**Methods:**

In this cross‐sectional study, between May and October 2022, women aged 30–60 years attending a tertiary care hospital in Gazipur district were approached for inclusion. Face‐to‐face interviews were conducted using a semistructured questionnaire. A total of 252 women were consecutively recruited within the study period. The self‐reported screening practice was recorded and verified by matching with identification numbers provided for screening by the hospital, and reasons for nonutilization were also collected.

**Results:**

Only 12 women (4.76%) had ever been screened for cervical cancer. Lower knowledge scores (OR: 0.26 and 95% CI: 0.08–0.95) were associated with higher odds of nonutilization of cervical cancer screening services on multivariable analysis. Despite high awareness of symptoms and risk factors, only 15.08% knew that screening prevents cancer. The main reasons for not getting screened were fear of pain (98.33%) and feeling shy (52.50%).

**Conclusion:**

Awareness‐increasing programs are recommended to improve the utilization of cervical cancer screening among women.

## 1. Introduction

In 2020, cervical cancer accounted for 604,127 cases and 341,831 deaths [1], making it the fourth most common cancer among women around the world [2]. Bangladesh experienced an estimated 156,775 cervical cancer cases and 108,990 cancer deaths during the same year [3]. The cancer is predicted to cause the deaths of more than 500,000 women by the year 2070 at the current rate [4]. Cervical cancer is caused by persistent human papillomavirus (HPV) infection, making it one of the few cancers that can be prevented through vaccination. Moreover, through screening and early detection, it can be cured completely through surgical options like radical hysterectomy [5].

With the availability of the HPV vaccine, governments are now planning to eradicate the disease in the next 2 decades [2] through nationwide vaccination of adolescent girls. Although vaccination could lead to the prevention of cervical cancer, it has been found to be less effective among women who are currently aged 27–45 years [6]. Therefore, screening and treatment of precancerous lesions remain the mainstay of prevention of cancer progression and associated deaths in a large number of women.

Although cervical cancer screening services are widely available in public health facilities, the utilization of the services is low in low‐ and middle‐income countries (LMICs). The screening coverage was found, on average, to be 19% in LMICs compared with 63% in high‐income countries [7]. In Bangladesh, the coverage rate was estimated to be 11.30% [3], a figure much lower than average estimates of LMICs. In general, the lack of knowledge and awareness has been identified as the most prevalent individual‐level barrier to the low utilization of screening programs in low‐resource settings [8]. Fear of pain, fear of being diagnosed with cancer, procedure cost, and embarrassment were some other reported reasons for not using screening services in LMICs [8–11].

In Bangladesh, cervical cancer screening through the visual inspection with acetic acid (VIA) method is provided from primary to tertiary level hospitals through trained health care providers [12]. However, the VIA service remained predominantly an opportunistic service [13], with screening uptake mostly based on doctors’ advice, referral, or awareness. Previous studies found a low level of utilization of cervical cancer screening in different areas of Bangladesh [14–16], with a regional variation in cervical cancer awareness and utilization of screening services within the country. However, the extent of cervical cancer screening uptake and its determinants have not been explored in Gazipur, a densely populated district located in the north‐central part of the country. Moreover, the HPV vaccination was started in Gazipur by the government through a pilot program in 2015, which can be expected to raise awareness among adult women regarding cervical cancer in this area [11]. Therefore, the present study aimed to examine the extent of screening service uptake among women attending a tertiary care public hospital in the Gazipur district. In addition, it tried to find out the factors influencing utilization and the reasons for nonutilization. Our study findings could shed light on regional variations in cervical screening utilization and aid in planning targeted health education campaigns to improve utilization.

## 2. Materials and Methods

### 2.1. Study Design, Setting, and Population

**Figure 1:**
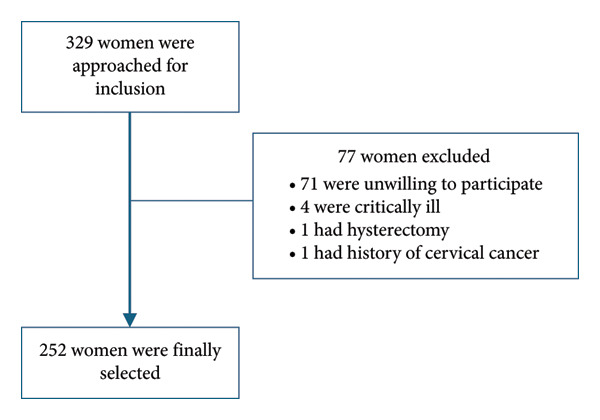
Participant selection diagram.

This was a cross‐sectional study conducted in the outpatient department (OPD) of the Department of Gynecology and Obstetrics at Shaheed Tajuddin Ahmed Medical College Hospital (STAMCH) in Gazipur, Bangladesh, between May 2022 and October 2022. The district has an estimated 5,263,450 population, with nearly half of them being women [17]. Out of the five primary‐level healthcare centers, only one provides cervical cancer screening services. Hence, most of the eligible women in this area have to take the service from STAMCH, which is the public tertiary care hospital in the district. We approached married women aged 30–60 years seeking health from STAMCH for inclusion. Women with a history of cervical cancer or hysterectomy, any critical illness, and those unwilling to participate were excluded from the study. The sample size was calculated based on the formula *n* = *z* ^2^pq/*d* ^2^. Taking *z* = standard normal deviates at 95% confidence interval = 1.96, *p* = proportion of cervical cancer screening utilization among women aged 30–65 years = 21.2% [18], *q* = 1–*p*, and *d* = margin of error = 5%, and the total sample size was calculated to be 257. Finally, within the stipulated time, a total of 252 participants were consecutively recruited. See Figure 1 for a flow diagram of participant selection.

### 2.2. Data Collection Technique

Face‐to‐face interviews were conducted for data collection by two trained data collectors using a semistructured pretested questionnaire (S1 File). They consecutively approached female patients and their attendees who come to the outdoors for consultation. However, they were approached only after they have completed their consultation with a physician at outdoors.

### 2.3. Research Instrument

A semistructured questionnaire was developed based on previously published articles [3, 5, 15, 19, 20] and discussion with experts. It was pretested on 20 women to assess its adaptability. The questionnaire consisted of sections on sociodemographic characteristics, menstrual health, reproductive health, and knowledge, attitude, and practice related to cervical cancer screening.

### 2.4. Scoring

The scoring pattern of the knowledge and attitude‐related questions is tabulated in the S2 File.

#### 2.4.1. Scoring of Knowledge‐Related Questions

A total of nine questions were asked to explore knowledge of cervical cancer and its screening covering symptoms, risk factors, prevention strategies, treatments, and cervical cancer screening frequency, age, and procedure. Scores were assigned as follows. A score of “1” was assigned for “yes” and “0” for “no” in questions with these two responses (K1, K2, and K3). The question on prevention methods (K4) had multiple‐choice options. However, a score of “1” was given for the selection of at least one choice, “0” was assigned for nonresponse. For the questions on the knowledge of whether cervical cancer can be treated or not, “1” was assigned for “yes” and “0” for either “no” or “do not know.” For treatment types (K6), “1” score was given for a choice of “surgery” and/or “radiotherapy,” “0” was assigned for selecting herbal remedies. For questions on frequency of screening (K7), “1” was assigned for the right answer only (every 5 years), the rest of the answers were assigned “0.” Although some places recommend screening every 3 years, we did not consider it a correct answer within the context of Bangladesh, where every 5 years is recommended and conventional knowledge. Similarly, for the question on who should be screened (K8), “1” was assigned for selecting “all women ≥ 25 years,” for the rest “0” was assigned. In procedures used in cervical screening, “1” was assigned for the selection of either “VIA” and/or “Pap smear,” and “0” was assigned for biopsy. Hence, the total score ranged between 0 and 9. A score of 80% or more (i.e., ≥ 8) was considered as having “good” knowledge; otherwise, “poor” knowledge.

#### 2.4.2. Scoring of Attitude‐Related Questions

A total of five attitude‐related questions were asked, covering consequences, risks, and role of screening in the prevention of cervical cancer as well as willingness to screen. Each attitude‐related question had a response comprising a three‐point Likert scale: “Agree,” “Neither agree nor disagree,” and “disagree.” A score of “3,” “2,” and “1” was assigned for each response, respectively. Therefore, the total score ranged between 5 and 15. A score of 80% or more (≥ 12) was considered “positive” attitude; otherwise, “negative” attitude.

#### 2.4.3. Screening Practice

For determining screening practice, a self‐reported question was asked on whether the participant ever screened for cervical cancer (i.e., used the service provided by the government for screening in the hospital). If the answer was yes, it was verified using the respective screening IDs provided by the hospital’s cervical cancer screening center. If the answer was “no,” a further question was asked to select from a list of eight reasons. These reasons were selected considering the context of the country, literature review [7], and discussion with experts.

### 2.5. Data Analysis

Data were analyzed using the statistical software RStudio 2023.12.1 + 402. As there were no missing data, no imputations were made. Descriptive statistics were expressed through frequency (proportion) and mean ± standard deviation for categorical and continuous variables, respectively. Pearson’s chi‐square test and Fisher’s exact test were used to assess associations between categorical factors and cervical cancer screening practice. Welch’s two‐sample *t*‐tests were used to explore differences in continuous variables across screening practice. Factors that became significant at bivariate screening were considered for exploration through logistic regression analysis. As one of the variables had complete separation across screening practice categories, we used Firth logistic regression [21] for the determination of independent factors behind “never screening” for cervical cancer. A *p* value of < 0.05 was considered significant for all statistical tests.

### 2.6. Ethical Considerations

Ethical approval was obtained from the Institutional Review Board of North South University. Permission was obtained from the hospital authorities. Written informed consent was obtained from all participants after explaining the study objectives. Participation was voluntary, and confidentiality was maintained throughout the study period. All procedures were conducted following the updated guidelines of the Declaration of Helsinki. The Strengthening the Reporting of Observational Studies in Epidemiology (STROBE) guidelines was used to report the findings of this study. The check list can be found in S3 File. The artificial intelligence model Claude Sonnet 4.0 was used to enhance the writing of the manuscript as the authors are not native English speakers.

## 3. Results

The average age of patients was 38.59 ± 6.91 years (±SD). The majority were aged 31–40 years (57.54%), were housewives (82.14%), and had primary (37.30%) or secondary (34.52%) education. Most had a low family income of 10,001–20,000 BDT (53.17%), lived in rural areas (83.33%), and got married before age 18 (86.90%) (see Table 1).

**Table 1:** Characteristics of the respondents.

Characteristic	*N* = 252^1^
Age (years)	38.59 (6.91)
Age groups (years)	
≤ 30 years	30 (11.90)
31–40 years	145 (57.54)
41–50 years	65 (25.79)
51–60 years	12 (4.76)
Education	
No formal education	42 (16.67)
Primary	94 (37.30)
SSC	87 (34.52)
HSC	10 (3.97)
Graduation and above	19 (7.54)
Occupation	
Housewife	207 (82.14)
Garment worker	22 (8.73)
Job holder	23 (9.13)
Husband’s education	
No formal education	34 (13.50)
Primary	71 (28.17)
SSC	103 (40.87)
HSC	17 (6.75)
Graduation and above	27 (10.71)
Husband’s occupation	
Business	59 (23.41)
Farmer	56 (22.22)
Garment worker	18 (7.14)
Job holder	57 (22.62)
Others	62 (24.60)
Monthly family income (BDT)	
≤ 10,000	70 (27.78)
10,001–20,000	134 (53.17)
20,001–30,000	24 (9.52)
> 30,000	24 (9.52)
Residence	
Rural	210 (83.33)
Urban	42 (16.67)
Age of marriage	
≤ 18 years	219 (86.90)
> 18 years	33 (13.10)
Number of family members	
More than four	144 (57.14)
Up to four	108 (42.86)
Comorbidities	
Absent	220 (87.30)
Present	32 (12.70)
Pain during menstruation	
Absent	238 (94.44)
Present	14 (5.56)
Regularity of menstrual cycle	
Irregular	23 (9.13)
Regular	229 (90.87)
Age at first pregnancy	
> 18	90 (35.71)
≤ 18	162 (64.29)
Number of pregnancies	
One	42 (16.67)
Two	84 (33.33)
More than two	126 (50.00)
Number of live births	
> 2	85 (33.73)
≤ 2	167 (66.27)
Number of MR or abortion	
More than one	14 (5.56)
None	207 (82.14)
One	31 (12.30)
Number of still births	
None	235 (93.25)
One or more	17 (6.75)
Hygiene product used	
Cloths and others	145 (57.54)
Sanitary napkin	107 (42.46)
Age at menarche (years)	
> 12	30 (11.90)
≤ 12	222 (88.10)
Use of family planning method	247 (98.02)
Oral contraceptives	226 (89.68)
Condom	28 (11.11)
Injectable contraceptives	45 (17.86)
Other methods	2 (0.79)
Knowledge score	7.96 (0.55)
Attitude score	12.56 (1.04)
Cervical cancer screening	
Ever screened	12 (4.76)
Never screened	240 (95.24)

^1^Mean (SD); *n* (%).

Table 2 shows that only 12 women (4.76%) had ever been screened for cervical cancer. Those ever screened had significantly higher mean knowledge scores of 8.25 versus 7.94 (*p* = 0.047). Attitude was not found to be associated with screening practice. Women whose husbands had “other” occupations were more likely to be screened (11.29%) compared with business (6.78%), farming (1.79%), garment work (none), and jobs (none) (*p* = 0.024). However, the use of family planning methods was also significantly associated with never being screened (4.05% vs. 40.00%, *p* = 0.019).

**Table 2:** Association of characteristics with cervical cancer screening experience.

Characteristic	Cervical cancer screening practice		*p* value
	Ever screened, *N* = 12^1^	Never screened, *N* = 240^1^	
Age (years)	42.42 (10.43)	38.40 (6.66)	0.212^2^
Education			0.758^3^
No formal education	2 (4.76)	40 (95.24)	
Primary	5 (5.32)	89 (94.68)	
SSC	4 (4.60)	83 (95.40)	
HSC	1 (10.00)	9 (90.00)	
Graduation and above	0 (0.00)	19 (100.00)	
Occupation			0.601^3^
Garment worker	0 (0.00)	22 (100.00)	
Housewife	12 (5.80)	195 (94.20)	
Job holder	0 (0.00)	23 (100.00)	
Husband’s education			0.432^3^
No formal education	0 (0.00)	34 (100.00)	
Primary	5 (7.04)	66 (92.96)	
SSC	6 (5.83)	97 (94.17)	
HSC	1 (5.88)	16 (94.12)	
Graduation and above	0 (0.00)	27 (100.00)	
Husband’s occupation			**0.024** ^3^
Business	4 (6.78)	55 (93.22)	
Farmer	1 (1.79)	55 (98.21)	
Garment worker	0 (0.00)	18 (100.00)	
Job holder	0 (0.00)	57 (100.00)	
Others	7 (11.29)	55 (88.71)	
Monthly family income (BDT)			0.599^3^
≤ 10,000	3 (4.29)	67 (95.71)	
10,001–20,000	7 (5.22)	127 (94.78)	
20,001–30,000	2 (8.33)	22 (91.67)	
> 30,000	0 (0.00)	24 (100.00)	
Residence			0.428^3^
Rural	9 (4.29)	201 (95.71)	
Urban	3 (7.14)	39 (92.86)	
Age of marriage			0.661^3^
≤ 18 years	10 (4.57)	209 (95.43)	
> 18 years	2 (6.06)	31 (93.94)	
Number of family members			0.932^4^
> 4	7 (4.86)	137 (95.14)	
Up to 4	5 (4.63)	103 (95.37)	
Any comorbidities			0.184^3^
Absent	9 (4.09)	211 (95.91)	
Present	3 (9.38)	29 (90.63)	
Pain during menstruation			> 0.999^3^
Absent	12 (5.04)	226 (94.96)	
Present	0 (0.00)	14 (100.00)	
Regularity of menstrual cycle			0.301^3^
Irregular	2 (8.70)	21 (91.30)	
Regular	10 (4.37)	219 (95.63)	
Age at first pregnancy			0.222^3^
> 18	2 (2.22)	88 (97.78)	
≤ 18	10 (6.17)	152 (93.83)	
Number of pregnancies			0.342^3^
More than two	7 (5.56)	119 (94.44)	
One	0 (0.00)	42 (100.00)	
Two	5 (5.95)	79 (94.05)	
Number of live births			0.756^3^
> 2	3 (3.53)	82 (96.47)	
≤ 2	9 (5.39)	158 (94.61)	
Hygiene product used			0.512^4^
Cloths and others	8 (5.52)	137 (94.48)	
Sanitary napkin	4 (3.74)	103 (96.26)	
Age at menarche (years)			0.370^3^
> 12	0 (0.00)	30 (100.00)	
≤ 12	12 (5.41)	210 (94.59)	
Use of family planning method			**0.019** ^3^
Yes	10 (4.05)	237 (95.95)	
No	2 (40.00)	3 (60.00)	
Number of MR or abortion			0.180^3^
More than one	2 (14.29)	12 (85.71)	
None	9 (4.35)	198 (95.65)	
One	1 (3.23)	30 (96.77)	
Number of still births			0.576^3^
None	11 (4.68)	224 (95.32)	
One or more	1 (5.88)	16 (94.12)	
Knowledge score	8.25 (0.45)	7.94 (0.55)	**0.047** ^2^
Knowledge categories			0.375^3^
Good	12 (5.48)	207 (94.52)	
Poor	0 (0.00)	33 (100.00)	
Attitude score	13.25 (1.60)	12.53 (1.00)	0.117^2^
Attitude categories			0.338^3^
Positive	10 (4.41)	217 (95.59)	
Negative	2 (8.00)	23 (92.00)	

*Note:* Significant *p* values at < 0.05 level were shown in bold.

^1^Mean (SD); *n* (%).

^2^Welch two‐sample *t*‐test.

^3^Fisher’s exact test.

^4^Pearson’s Chi‐squared test.

On multivariable analysis, using any family planning method remained significantly associated with higher odds of never being screened (OR: 1.21, 95% CI: 1.58–8.81, and *p* = 0.018) compared with not using a method. Lower knowledge scores were significantly associated with higher odds of never being screened (OR: 0.26, 95% CI: 0.08–0.95, and *p* = 0.042) for each unit increase in knowledge score (see Table 3).

**Table 3:** Logistic regression analyses exploring factors associated with nonutilization cervical cancer screening service.

Characteristic	Univariate			Multivariate		
	OR^1^	95% CI^1^	*p* value	OR^1^	95% CI^1^	*p* value
Husband’s occupation						
Business	Ref	—	—	Ref	—	—
Farmer	3.00	0.53–37.69	0.222	2.47	0.41–26.01	0.336
Garment worker	3.00	0.29–404.69	0.407	2.12	0.20–289.24	0.591
Job holder	9.32	0.96–1246.49	0.055	8.01	0.81–1075.05	0.080
Others	0.60	0.16–1.99	0.408	6.21	0.15–2.32	0.477
Use of family planning method						
Yes	16.15	2.47–93.09	**0.006**	1.21	1.58–8.81	**0.018**
No	Ref	—	—	Ref	—	—
Knowledge score	0.31	0.10–1.00	0.051	0.26	0.08–0.95	**0.042**

*Note:* Significant *p* values at < 0.05 level were shown in bold.

^1^OR = odds ratio, CI = confidence interval.

Table 4 shows the major reasons for not getting screened were fear of pain (98.33%), feeling shy (52.50%), and perceiving no necessity as they felt healthy (9.17%). Lack of information (2.50%) and perceiving the test as expensive (1.25%) were minor reasons.

**Table 4:** Reasons for nonutilization of cervical cancer screening services.

Characteristic^∗^	*N* = 240^1^
Fear of pain	236 (98.33)
Feels shy	126 (52.50)
Feels healthy and does not see the necessity of vaccination	22 (9.17)
Not informed about the cervical screening program	6 (2.50)
Thinks the test is expensive	3 (1.25)
Husband would disagree	1 (0.42)
Undecided about screening	1 (0.42)

^1^ *n* (%).

^∗^Multiple responses were considered.

The responses to different knowledge and attitude‐related questions are listed in Supporting Tables 1 and 2. Nearly all could identify vaginal bleeding (99.60%), foul smell (99.21%), and multiple sexual partners (99.21%) as symptoms and risk factors, respectively. However, only 15.08% knew screening prevents cervical cancer, and 75.40% did not think it could be treated, despite 85.32% recognizing radiotherapy as a treatment option. While 90.08% correctly stated the recommended 5‐year screening interval and 99.21% knew all women ≥ 25 years should be screened, awareness of screening procedures like Pap smear (0.79%) was very low compared with visual inspection with acetic acid (98.81%). Regarding attitudes, although most neither agreed nor disagreed that cervical cancer causes death (92.86%) or that any woman can acquire it (73.81%), 48.81% agreed screening is preventive. Encouragingly, willingness to get screened was high at 86.51% regardless of fee and 92.46% if free, suggesting costs and lack of access could be barriers.

## 4. Discussion

This cross‐sectional study explored the extent of cervical cancer screening utilization and associated factors among women aged 30–60 years attending a tertiary care hospital in Gazipur, Bangladesh. The key findings were the very low screening rate of only 4.76%, lower knowledge scores being associated with never being screened, and the use of family planning methods also being associated with never being screened. Fear of pain and shyness were the main reasons cited by the participants for nonutilization of cervical cancer screening service.

The screening rate of 4.76% is alarmingly low compared with the estimated national average of 11.30% [3] and far below the rates in high‐income countries (over 60%) [7]. It also falls far below the expected coverage of 40% of the target population in Bangladesh [12] and the World Health Organization recommended target of 70% [22]. Although our study was hospital based, as the majority of the women in this region were taking the service from our study center, the low utilization rate gives a good reflection of the community coverage. Hence, the utilization rate found in our study underscores major gaps in cervical cancer prevention efforts by the health authorities in this industrialized district of Bangladesh. A region‐focused approach to raise awareness regarding cervical cancer screening service availability and the benefits of using the service should be considered.

We found that the lower the knowledge scores, the higher the odds of never being screened. These findings relay a similar association observed in the community‐based study in Dhaka [15], the Capital of Bangladesh. Likewise, studies conducted in other countries like Ethiopia [23–25] and Iran [26] have also repeatedly shown this association, highlighting the pivotal role of educating women about cervical cancer screening.

While awareness of cervical cancer symptoms, risk factors like multiple partners, and the need for all women over 25 to be screened was quite high, knowledge of screening procedures like the Pap smear was extremely poor. The latter finding could be expected as the screening method used in Bangladesh is VIA, and hence, participants could be unaware of the Pap smear method. Disturbingly, most women did not think cervical cancer was treatable despite recognizing radiotherapy as a treatment option. One possibility could be that respondents linked radiotherapy with cancer in general, as the term “radiotherapy” is commonly heard as a treatment for malignancies, while “surgery” is less often heard as a treatment option for malignancies in general. Perhaps this is the reason for choosing radiotherapy as a therapeutic option by the participants.

Awareness that screening can actually prevent cervical cancer was lacking in over 80% of the participants. Studies have shown that the lack of knowledge and awareness about cervical cancer and its screening program is the most common reason for the low utilization of screening services in LMICs [8, 10]. As many potentially treatable cases are missed due to a lack of screening, this crucial gap in knowledge could be linked to low utilization and, thereby, to the increasing morbidity and mortality associated with cervical cancer in Bangladesh [3, 14].

Intriguingly, using family planning methods was strongly associated with never being screened in this study. One could hypothesize that women relying on family planning may have lower risk perceptions for gynecological cancers. One study found that less than 10% of married women who lived apart from their spouse ever had extramarital sex [27], and in the presence of a spouse, the practice would be much lower. This fact, when considered in the context that more than 98% of our participants were using one of the family planning methods, plausibly gave our participants a sense of security from the risk of cervical cancer.

Considering the experiences of experts and from piloting and reviewing the literature, we enlisted several reasons for the nonutilization of screening services to be explored among our participants. These were in addition to the questions for awareness and attitude. The reasons vary widely in distribution throughout the world [8, 10]. However, we observed that fear of pain and shyness were cited as the top reasons for not getting screened by the vast majority of participants in this study. Fear of pain has been reported as one of the reasons by women in Malawi [28] and South Africa [29] as well. Although none of the cervical cancer screening methods should cause pain, some women might experience pain due to the inexperienced handling of the speculum [30]. VIA, the method commonly used in our settings, is usually well received by the women [31]. Hence, the fear of pain might be expressing the fear of discomfort often felt by women undergoing cervical cancer screening [30]. Likewise, feeling embarrassed is often described as one of the main reasons for nonparticipation in screening programs. For example, a sample of Bruneian women [9] shared such a feeling. It has also been reported by many women who have undergone the procedure [30]. Hence, Qayum and colleagues [15] have highlighted the necessity of ensuring privacy during the screening procedure.

A small proportion of participants also perceived no necessity of cervical screening as they felt healthy. This probably indicates a lack of awareness about the purpose of cancer screening. Lack of information about the service and cost, although cited as the main reason for nonutilization [10] in other studies, appeared as a minor reason in our exploration.

Unlike findings from Iran [26], Tanzania [32], or Ethiopia [24, 25, 33], we did not find any association of low utilization of screening services with age, education, monthly income, parity, or residence, which probably could be a limitation due to a low proportion of women having screening services. However, this may also indicate that the low utilization behavior was similar irrespective of the sociodemographic and reproductive characteristics of women in the Gazipur district.

Based on the findings of the questions, we noted that a majority of participants believed that cervical cancer is untreatable, and a few mentioned herbal remedies. These findings suggest a need for stronger public education emphasizing that cervical cancer is treatable, especially if detected early. Recommended medical treatments, for example, surgery, radiotherapy, and chemotherapy, should be clearly communicated through health messaging. Also, knowledge appears limited to VIA, with almost no recognition of Pap smears or HPV testing. While this could be due to the availability of VIA as the most accessible and practiced option in this context, the health education materials should still present a full picture of available screening methods, particularly in better‐resourced or urban settings. Also, the belief that only certain groups (e.g., sex workers) are at risk persists. Educational strategies must clarify that all women ≥ 25 years are at risk and should be screened. The fatal consequences of untreated cervical cancer should also be more strongly emphasized to convey urgency and relevance.

Despite the knowledge and practice gap and the reasons behind it, an encouraging finding was that nearly half the participants agreed cervical cancer screening is preventive, and more than four‐fifths were willing to get screened despite fees, a figure concordant with the pooled acceptance of screening service of 89.5% across the globe [34]. This positive attitude could be leveraged by the health authorities to motivate women to come to the screening centers and increase the screening coverage in this region.

The study’s strengths include being one of the earliest to explore cervical cancer utilization practice and reasons behind nonpractice in Gazipur, Bangladesh. Moreover, the utilization of cervical cancer screening was confirmed through records of the patients. Limitations are the relatively small sample size from a single tertiary care center and the consecutive sampling approach. However, women coming from the community to consult for their problems in the outdoors and indoors of hospitals have an inherent randomness in the sense that for any consecutive sample of them would be random, and their reasons for consultation do not follow any specific pattern and are not related to cervical cancer screening. Also, the accompanying female attendees are apparently healthy. Hence, a consecutively taken sample from both female patients and attendees could be considered representative of the community living in Gazipur. The potential recall bias associated with self‐reporting of practice was minimized through confirmation of screening from records for those giving an affirmative answer. For participants giving a negative answer, the possibility of recall bias was expected to be low because of the sensitivity of the method used in the screening process. To avoid self‐selection bias, we ensured consecutive sampling of participants coming to the outdoors irrespective of their reasons and that they were not conveniently included. However, the possibility of a desirability bias, demonstrated through a high proportion of correct answers despite poor practice, could not be avoided.

In conclusion, this study flags an alarmingly low cervical cancer screening rate in an area of Bangladesh and highlights critical gaps in knowledge that need to be addressed through educational interventions to improve screening uptake. Innovative measures to alleviate fear, embarrassment, and other barriers are essential for an effective screening program. On this point, patient education programs explaining the objectives and process of screening could be arranged, and screening provisions maintaining strict privacy by female health workers should be ensured.

## Conflicts of Interest

The authors declare no conflicts of interest.

## Funding

The study did not receive any funds.

## Supporting Information

Additional supporting information can be found online in the Supporting Information section.

## Supporting information


**Supporting Information 1** Supporting Table 1. Participant’s responses to questions related to knowledge of cervical cancer screening.

**Supporting Information 2** Supporting Table 2. Participant’s responses to questions related to attitude towards cervical cancer screening.

**Supporting Information 3** S1 File. Questionnaire.

**Supporting Information 4** S2 File. Knowledge and Attitude Question Scoring Codebook.

**Supporting Information 5** S3 File. STROBE Checklist for Cross‐sectional Study.

## Data Availability

The data that support the findings of this study are available from the corresponding author upon reasonable request.
