# Supplementary material for: Factors Associated With Low Utilization of Cervical Cancer Screening Services in Gazipur, Bangladesh
Source: Obstet Gynecol Int. 2025 Dec 22;2025:4476955. doi: 10.1155/ogi/4476955 (PMC12767436; doi:10.1155/ogi/4476955)
Supplement: Supplementary file 1 — Supporting Information 1 Supporting Table 1. Participant’s responses to questions related to knowledge of cervical cancer screening. [file OGI-2025-4476955-s001.docx]

**Supplementary Table 1. Participants’ responses to questions related to knowledge of cervical cancer screening (n=252)**

| **Characteristic** | n (%) |
| --- | --- |
| Vaginal bleeding is a symptom of cervical cancer | 251 (99.60) |
| Vaginal foul smell is a symptom of cervical cancer | 250 (99.21) |
| Multiple sexual partners are a risk factor | 250 (99.21) |
| Prevention methods for cervical cancer |  |
| Avoiding multiple sexual partners | 206 (81.75) |
| Cervical cancer screening | 38 (15.08) |
| Avoiding early sexual intercourse | 4 (1.59) |
| Quitting smoking | 4 (1.59) |
| Cervical cancer can be treated |  |
| Don't know | 34 (13.49) |
| No | 190 (75.40) |
| Yes | 28 (11.11) |
| Treatment types available for cervical cancer |  |
| Herbal remedies | 6 (2.38) |
| Radiotherapy | 215 (85.32) |
| Surgery | 31 (12.30) |
| How frequently should one be screened for cervical cancer? |  |
| Do not know | 16 (6.35) |
| Every five years | 227 (90.08) |
| Every three years | 8 (3.17) |
| Once a year | 1 (0.40) |
| Who should be screened? |  |
| All women of >=25 years | 250 (99.21) |
| Prostitutes only | 2 (0.79) |
| What procedures are used in cervical cancer screening? |  |
| VIA | 249 (98.81) |
| Pap smear | 2 (0.79) |
| Both VIA and pap smear | 1 (0.40) |
