# Supplementary material for: Factors Associated With Low Utilization of Cervical Cancer Screening Services in Gazipur, Bangladesh
Source: Obstet Gynecol Int. 2025 Dec 22;2025:4476955. doi: 10.1155/ogi/4476955 (PMC12767436; doi:10.1155/ogi/4476955)
Supplement: Supplementary file 2 — Supporting Information 2 Supporting Table 2. Participant’s responses to questions related to attitude towards cervical cancer screening. [file OGI-2025-4476955-s002.docx]

**Supplementary Table 2. Participants’ responses to questions related to attitude towards cervical cancer screening (n=252)**

| **Characteristic** | **n (%)** |
| --- | --- |
| Carcinoma of the cervix is the cause of death |  |
| Agree | 13 (5.16) |
| Disagree | 5 (1.98) |
| Neither agree nor disagree | 234 (92.86) |
| Any woman can acquire cervical cancer |  |
| Agree | 65 (25.79) |
| Disagree | 1 (0.40) |
| Neither agree nor disagree | 186 (73.81) |
| Screening helps in the prevention of cervical cancer |  |
| Agree | 123 (48.81) |
| Neither agree nor disagree | 129 (51.19) |
| Willing to screen regardless of fee |  |
| Agree | 218 (86.51) |
| Neither agree nor disagree | 34 (13.49) |
| Willing to screen if cancer screening is free |  |
| Agree | 233 (92.46) |
| Neither agree nor disagree | 19 (7.54) |
