# Supplementary material for: Factors Associated With Low Utilization of Cervical Cancer Screening Services in Gazipur, Bangladesh
Source: Obstet Gynecol Int. 2025 Dec 22;2025:4476955. doi: 10.1155/ogi/4476955 (PMC12767436; doi:10.1155/ogi/4476955)
Supplement: Supplementary file 3 — Supporting Information 3 S1 File. Questionnaire. [file OGI-2025-4476955-s003.docx]

**QUESTIONNAIRE**

| Name |  | UID |  |
| --- | --- | --- | --- |
| Address |  | Phone no |  |

| **Section-A: Socio-demographic information** | | |
| --- | --- | --- |
| Q-No | Question | Answer |
| SD-1 | Participant’s Age |  |
| SD-2 | Educational Qualification | 1. No Education  2. Up to Primary  3. Up to SSC  4. Up to HSC  5. Hons or above |
| SD-3 | Occupation |  |
| SD-4 | Husband's educational qualification | 1. No Education  2. Up to Primary  3. Up to SSC  4. Up to HSC  5. Hons or above |
| SD-5 | Husband's occupation |  |
| SD-6 | Household income |  |
| SD-7 | Residence | 1. Rural / Village  2. Suburban  3. Urban /City |
| SD-8 | Number of family members |  |
| SD-9 | What is the age that you get married |  |
| SD-10 | Do you have any chronic disease? (e.g., diabetes, hypertension, etc.) | 1. Yes 2. No |
| **Section-B: Menstrual Health** | | |
| MH-11 | Do you feel any pain during menstruation? | 1. Yes 2. No |
| MH-11a | If yes, what is the severity of the pain that you feel? | 1. Mild 2. Moderate 3. Severe |
| MH-12 | Menstrual cycle | 1. Regular 2. Irregular 3. Don’t know |
| MH-13 | Age at menarche in years |  |
| MH-14 | How many days did your last period last? |  |
| MH-15 | What type of hygiene products do you use during menstruation?  (Multiple responses allowed) | 1. Cloth 2. Sanitary pads 3. Menstrual cups 4. Others (Specify…) |
| **Section-C: Reproductive Health** | | |
| RH-1 | Age of first pregnancy |  |
| RH-2 | How many times did you get pregnant? |  |
| RH-3 | Number of live births |  |
| RH-4 | Number of MR/Abortion |  |
| RH-5 | Number of stillbirths |  |
| RH-6 | What type of family planning method do you use?  (Multiple choices allowed) | 1. Pill 2. Condom 3. Injection 4. None 5. Others (Specify…) |
| **Section-D: Knowledge, Attitude and Practice towards cervical cancer screening** | | |
| **K-1** | Vaginal bleeding is a symptom of cervical cancer | Yes  No |
| **K-2** | Vaginal foul smell is symptom of cervical cancer | Yes  No |
| **K-3** | Multiple sexual partners are a risk factor | Yes  No |
| **K-4** | Prevention methods for cervical cancer |  |
|  | K-4a. Avoiding multiple sexual partners | Yes  No |
|  | K-4b. Avoiding early sexual exposure | Yes  No |
|  | K-4c. Quitting smoking prevents cervical cancer | Yes  No |
|  | K-4d. HPV vaccination prevents cervical cancer | Yes  No |
|  | K-4e. Screening prevents cervical cancer | Yes  No |
| **K-5** | Cervical cancer can be treated | Yes  No  Do not know |
| **K-6** | Treatment types available for cervical cancer | Herbal remedies  Surgery  Radiotherapy  Chemotherapy |
| **K-7** | How frequently should one be screened for cervical cancer? | Once a year  Every three years  Every five years  Any other  Don’t know |
| **K-8** | Who should be screened? | Women aged 25 and above  Older women only  Women with high-risk sexual behavior  Others (Specify…) |
| **K-9** | What procedures are used in cervical cancer screening?  (Multiple choices allowed) | VIA  Pap smear  Biopsy  Don’t know |
| **A-1** | Carcinoma of the cervix is the cause of death | Agree  Neither agree nor disagree  Disagree |
| **A-2** | Any woman can acquire cervical cancer | Agree  Neither agree nor disagree  Disagree |
| **A-3** | Screening helps in the prevention of cervical cancer | Agree  Neither agree nor disagree  Disagree |
| **A-4** | You are willing to undergo cervical cancer screening | Agree  Neither agree nor disagree  Disagree |
| **A-5** | If screening for cancer is free, will you be screened? | Agree  Neither agree nor disagree  Disagree |
| **A-6** | Screening is unnecessary if I feel healthy  (This question was added by the reviewer’s suggestion for future users.) | Agree  Neither agree nor disagree  Disagree |
| **P-1** | Have you ever been screened for cervical cancer? | Yes  No |
| **P-1.2** | If yes, then how many times screened | Once  More than once |
| **P-2** | Reason for not being screened  (Multiple choices allowed) | It may be painful  I feel shy  I am healthy  My husband would not agree  A screening test reveals cancer  It is expensive  I am not informed  I have not decided |
