# Supplementary material for: Factors Associated With Low Utilization of Cervical Cancer Screening Services in Gazipur, Bangladesh
Source: Obstet Gynecol Int. 2025 Dec 22;2025:4476955. doi: 10.1155/ogi/4476955 (PMC12767436; doi:10.1155/ogi/4476955)
Supplement: Supplementary file 4 — Supporting Information 4 S2 File. Knowledge and Attitude Question Scoring Codebook. [file OGI-2025-4476955-s004.docx]

**S2 File. Scoring scheme for knowledge and attitude questions related to cervical cancer screening**

The scores counted for each response are presented in parentheses

| **Knowledge related questions** | | |
| --- | --- | --- |
| **K-1** | Vaginal bleeding is a symptom of cervical cancer | Yes (1)  No (0) |
| **K-2** | Vaginal foul smell is a symptom of cervical cancer | Yes (1)  No (0) |
| **K-3** | Multiple sexual partners are a risk factor | Yes (1)  No (0) |
| **K-4** | Prevention methods | Avoiding multiple sexual partners prevents cervical  Avoiding early sexual intercourse  Quitting smoking prevents cervical cancer  HPV vaccination prevents cervical cancer  Screening prevents cervical cancer  (One or more response = 1; No response = 0) |
| **K-5** | Knowing cancer of the cervix can be treated | Yes (1)  No (0)  Do not know (0) |
| **K-6** | Treatment type | Herbal remedies (0)  Surgery (1)  Radiotherapy (1)  (Either surgery or radiotherapy = 1; herbal remedies = 0) |
| **K-7** | Frequency of screening | Once a year (0)  Every three years (0)  Every five years (1)  Any other (0)  Do not know (0) |
| **K-8** | Who should be screened? | All Women ≥ 25 years (1)  Prostitutes only (0)  Elderly women (>65 years) (0)  Others (0) |
| **K-9** | Procedures used in cervical cancer screening | VIA (1)  Pap smear (1)  Biopsy (0)  (VIA or pap smear = 1; Biopsy = 0) |
| **A-1** | Carcinoma of the cervix is the cause of death | Agree (3)  Neither agree nor disagree (2)  Disagree (1) |
| **A-2** | Any woman can acquire cervical cancer | Agree (3)  Neither agree nor disagree (2)  Disagree (1) |
| **A-3** | Screening helps in the prevention of cervical cancer | Agree (3)  Neither agree nor disagree (2)  Disagree (1) |
| **A-4** | Willingness for screening | Agree (3)  Neither agree nor disagree (2)  Disagree (1) |
| **A-5** | If screening for cancer is free, will you be screened? | Agree (3)  Neither agree nor disagree (2)  Disagree (1) |
| A-6 | Screening is unnecessary if I feel healthy  (This question was added by the reviewer’s suggestion for future users.) | Agree (1)  Neither agree nor disagree (2)  Disagree (3) |
